# Exoscopic resection of a parasagittal atypical meningioma

**DOI:** 10.3171/2023.10.FOCVID23164

**Published:** 2024-01-01

**Authors:** Alejandro Carrasquilla, Artur Zgurov, Mira Salih, Chi Le, Stavros Matsoukas, Rui Feng, Alexander J Schupper, Constantinos Hadjipanayis

**Affiliations:** 1 Department of Neurosurgery, Icahn School of Medicine at Mount Sinai, New York, New York; and; 2 Department of Neurosurgery, University of Pittsburgh Medical Center, Pittsburgh, Pennsylvania

**Keywords:** 3D exoscope, bilateral parasagittal atypical meningioma, surgical efficiency and ergonomics, craniotomy, brain tumor, superior sagittal sinus, WHO grade II, neuronavigation

## Abstract

This video demonstrates use of the Synaptive 3D exoscope to enhance complex meningioma resection. The patient was a 58-year-old female who presented with new-onset seizures. Workup revealed a parasagittal meningioma over the bilateral cortices. She was started on 750 mg of Keppra twice daily and tapered dexamethasone and discharged. MR venography demonstrated segmental occlusion of the superior sagittal sinus. She then underwent a diagnostic angiogram and tumor Onyx embolization of the bilateral middle meningeal artery feeders. She then underwent a craniotomy for meningioma resection using 3D exoscope guidance. She awoke with a stable examination in the intensive care unit and worked with physical therapy on postoperative day 1.

The video can be found here: https://stream.cadmore.media/r10.3171/2023.10.FOCVID23164

## Transcript

We will be presenting the exoscopic resection of a parietal parasagittal meningioma from the Department of Neurosurgery at Mount Sinai in New York City.

### 0:30 Patient Presentation.

Patient was a 58-year-old female who presented with new-onset seizures. Workup revealed a parasagittal meningioma over the bilateral cortices. She was started on Keppra 750 BID and dex taper. MRV demonstrated segmental occlusion of superior sagittal sinus. She underwent angiogram and tumor Onyx embolization of bilateral middle meningeal artery feeders. She then underwent craniotomy for meningioma resection using 3D exoscope guidance.

### 1:00 Positioning and Preparation.

Neurophysiology monitoring was utilized during surgery to track motor evoked potentials, somatosensory evoked potentials, and electroencephalography. The head was placed in three-pin Mayfield fixation and flexed to allow for exposure of the vertex. Synaptive neuronavigation was registered successfully and used to design the bicoronal incision. Here you can see the ergonomics of using the exoscope in this case. The exoscope helped our comfort, efficiency, and efficacy through this operation.

### 1:29 Surgical Procedure.

The scalp was then incised and mobilized as a single myocutaneous flap. Neuronavigation was used to design the craniotomy. A 5-mm cutting burr was used to create burr holes on the periphery, and a matchstick bit was used to carve wider slots over the sagittal sinus in order to optimize safe detachment of the bone flap. The bone flap was then carefully elevated from the dura mater.

### 1:51 Removal of Left Parafalcine Tumor.

The dura was first incised over the left lateral edge of the tumor. The brain-tumor interface was identified, and bipolar electrocautery was used to shrink tumor away from the brain surface. Cottonoids were used to cover and preserve the brain surface while suction and electrocautery were used to dissect the tumor. Of note, the tumor was quite fibrous and tough and also invaded into the brain. There were slight changes in motor evoked potentials on the left side consistent with right lower-extremity changes. This coincided with where the bulk of the tumor was during the surgery and its highly invasive nature found during surgery. Once separated from the brain, the tumor could be cut and debulked in larger portions.

### 2:40 Removal of Right Parafalcine Tumor.

We then incised dura along the right contralateral edge of the tumor, and tumor was separated in a similar fashion. A combination of electrocautery, scissors, and ultrasonic aspirator were used for tumor debulking. Here, tumor is being dissected off the falx. The midline portion of the superior sagittal sinus was completely occluded by tumor. This portion could therefore be fully resected. Surgicel was used to line the resection cavity, and fibrin glue was placed over the cut ends of the superior sagittal sinus as a precaution.

### 3:30 Postoperative Course.

She awoke from surgery with a stable exam and worked with physical therapy on postoperative day 1. Pathology resulted as atypical meningioma WHO grade II.

WHO grade II atypical meningiomas make up 20%–35% of meningiomas.^1,2^ In a study of 165 patients, univariate and multivariate analyses showed that male sex, meningioma grade, and surgical time more than 232 minutes were associated with decreased progression-free survival (PFS).^3^ In a series of 108 atypical meningioma patients treated at a single institution with confirmed Simpson grade I gross-total resection, 28% recurred after an average follow-up of 39 months.^4^ Obtaining a gross-total resection via surgery remains the best first-line treatment toward overall survival; however, in most patients, adjuvant radiotherapy is also recommended.^5^

## Disclosures

Dr. Hadjipanayis reported personal fees from Hemerion Therapeutics, Stryker Corp., Synaptive Medical, and Integra outside the submitted work.

## Author Contributions

Primary surgeon: Hadjipanayis. Assistant surgeon: Carrasquilla, Matsoukas, Feng. Editing and drafting the video and abstract: Carrasquilla, Zgurov, Salih, Feng, Schupper, Hadjipanayis. Critically revising the work: Carrasquilla, Salih, Feng, Schupper, Hadjipanayis. Reviewed submitted version of the work: Carrasquilla, Salih, Le, Matsoukas, Feng, Schupper, Hadjipanayis. Approved the final version of the work on behalf of all authors: Carrasquilla. Supervision: Carrasquilla, Hadjipanayis.

## Supplemental Information

### Patient Informed Consent

Patient consent was obtained for surgery and for collection of video and photography of the procedure. Consent for publication was not obtained as the patient was deceased prior to the preparation of the submission.
